# Molecular Epizootiology of *Toxoplasma gondii* and *Cryptosporidium parvum* in the Eastern Oyster (*Crassostrea virginica*) from Maine (USA)

**DOI:** 10.3390/pathogens8030125

**Published:** 2019-08-13

**Authors:** Nicholas D. Marquis, Theodore J. Bishop, Nicholas R. Record, Peter D. Countway, José A. Fernández Robledo

**Affiliations:** 1 Bigelow Laboratory for Ocean Sciences, Boothbay, ME 04544, USA; 2 Southern Maine Community College, South Portland, ME 04106, USA

**Keywords:** *Cryptosporidium*, epizootiology, Maine, oysters, qPCR, *Toxoplasma*

## Abstract

Shellfish are known as a potential source of *Toxoplasma gondii* (responsible for toxoplasmosis), and *Cryptosporidium parvum*, which is one of the major causes of gastroenteritis in the world. Here we performed a comprehensive qPCR-based monthly survey for *T. gondii* and *C. parvum* during 2016 and 2017 in oysters (*Crassostrea virginica*) (n = 1440) from all six sites along the coast of Maine (USA). Pooled samples (mantle, gills, and rectum) from individual oysters were used for DNA extraction and qPCR. Our study resulted in detections of qPCR positives oysters for *T. gondii* and *C. parvum* at each of the six sites sampled (in 31% and 10% of total oysters, respectively). The prevalence of *T. gondii* was low in 2016, and in September 2017 several sites peaked in prevalence with 100% of the samples testing positive. The prevalence of *C. parvum* was very low except in one estuarine location (Jack’s Point) in June 2016 (58%), and in October of 2016, when both prevalence and density of *C. parvum* at most of the sampling sites were among the highest values detected. Statistical analysis of environmental data did not identify clear drivers of retention, but there were some notable statistically significant patterns including current direction and nitrate along with the *T. gondii* prevalence. The major *C. parvum* retention event (in October 2016) corresponded with the month of highest dissolved oxygen measurements as well as a shift in the current direction revealed by nearby instrumentation. This study may guide future research to locate any contributing parasite reservoirs and evaluate the potential risk to human consumption.

## 1. Introduction

*Toxoplasma gondii* and *Cryptosporidium parvum* are two cosmopolitan protozoan parasites relevant to public health [1]. The definitive hosts for *T. gondii* are felines, including both feral and pet domestic cats, and others such as bobcats and pumas. Carnivores acquire *T. gondii* from consuming infected prey; though other animals (including humans) can incidentally acquire the parasite through foodborne pathways, and in the case of humans, consuming undercooked meat, contaminated plants and water, contact with cat litter, and blood transfusions, are additional sources of infection [2]. Estimates show that *T. gondii* infects 2 billion people worldwide with most infected individuals remaining asymptomatic. Despite the lack of symptoms in those with good health, *T. gondii* is a significant threat to AIDS patients and otherwise immunosuppressed patients, as well as pregnant women [3]. Recently, *T. gondii* has attracted public attention due to numerous publications linking toxoplasmosis to mental disorders [4,5,6,7] and problematic behaviors (e.g., increased likelihood of risky road behaviors that have been associated with traffic accidents and ‘risk taking’ related to business decisions) [8,9]. Reports exist of *T. gondii* in marine mammals including sea otters (*Enhydra lutris*) [10], beluga whales (*Delphinapterus leucas*) [11]. *T. gondii* oocysts isolated from experimentally exposed mussels are associated with morbidity and mortality in murine bioassays [12], further supporting the basis for public health concern. Additionally, studies in Taiwan revealed that consuming clams and having a cat in the household are two independent risk factors for acute toxoplasmosis [13]. *Cryptosporidium parvum* is a pathogen commonly found in domestic ruminants. Humans and wild animals are typically infected by ingesting water or grass contaminated with oocysts. Cryptosporidiosis symptoms include watery diarrhea, dehydration, weight loss, stomach cramps or pain, fever, nausea, and vomiting, and it is a severe threat to children under four in underdeveloped countries [14,15]. Reported *Cryptosporidium* outbreaks in the USA have been associated with contamination of drinking water, with no outbreaks yet linked to consuming contaminated mollusks [14].

**Figure 1:**
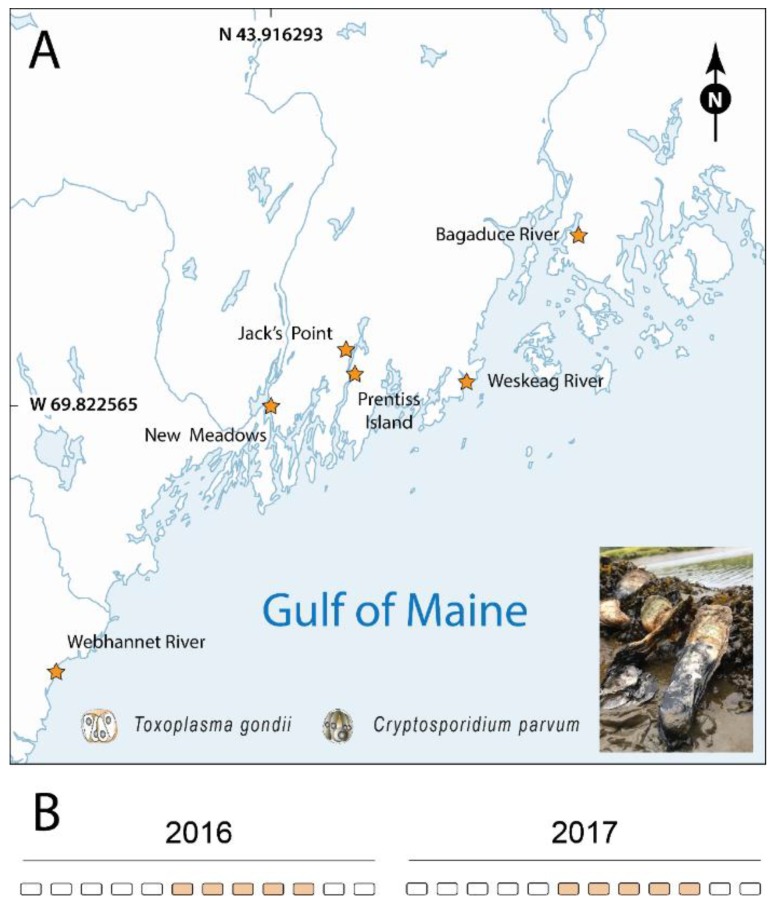
Oyster collection sites in Gulf of Maine tributaries (stars). (**A**) Adult eastern oysters (*Crassostrea virginica*, inset photograph) were collected between June and October during 2016 and 2017 from shellfish farmers located at six oyster leases along the coast of the Gulf of Maine (USA). (**B**) Sampling calendar for both years of the project.

Oysters are considered ecosystem engineers. Through their filter-feeding, they influence the estuarine water quality and turbidity, provide habitat for other species, and they are a significant food resource for humans [16,17]. Because bivalves are capable of filtering volumes of water, they are particularly exposed to environmental contaminants and accumulate a wide range of terrene and marine pollutants, including heavy metals, hydrocarbons, chlorinated hydrocarbons, microplastics, radionuclides, and parasites from human and animal waste; consequently, they are considered as excellent beacons for monitoring the health of estuarine ecosystems [14,18,19,20]. The coast of Maine (USA) has long been known as an ideal location for bivalve cultivation due to the pristine watersheds that empty into miles of protected and undeveloped shoreline [21]. Historically, the low occurrence of shellfish die-offs due to disease-causing agents (reviewed in [22]) has also nurtured the expansion of the aquaculture industry in the area. Although most studies of pathogens in oysters have focused on bivalve disease-causing agents, both *T. gondii* and *C. parvum* have been reported in oysters from Maine using PCR-based diagnosis with maximum prevalences of 14 and 39%, respectively [23]. In the current study, we expanded on these previous findings and performed an extensive quantitative PCR-based survey for *T. gondii* and *C. parvum* during 2016 and 2017 at six locations in coastal Maine (Figure 1).

## 2. Results

### 2.1. Toxoplasma gondii Prevalence and Densities

**Figure 2:**
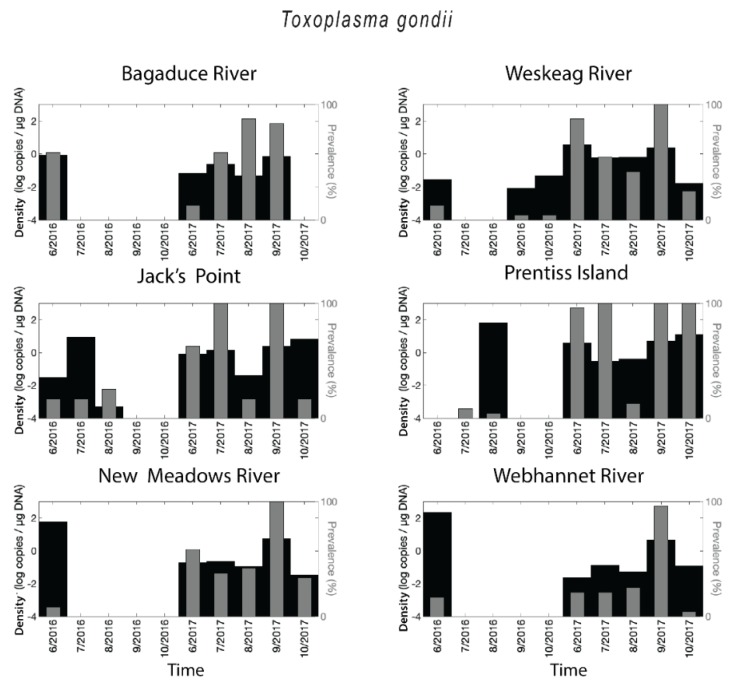
Spatial-temporal patterns for *Toxoplasma gondii*. Retention density (as log of copies per µg DNA, shown as black bars) and parasite prevalence (as a percentage of oysters infected, shown as grey bars) for each sampling site, for each sampling period.

*Toxoplasma gondii* DNA was identified at each of the six sites tested with the highest prevalence corresponding to samples from Jack’s Point and Prentiss Island in the Damariscotta River Estuary (DRE) in 2017 (Figure 2). All sites showed distinct differences in prevalence between 2016 and 2017, with much higher prevalence in 2017. Prevalence values in the Webhannet River remained below 60% during the two-year survey except for the peak observed in September 2017. When the parasite was present, *T. gondii* densities (target copies/µg DNA) were in the range of less than 0.001 up to 219 copies/µg DNA. The shift between 2016 and 2017 was also apparent in density values, but there were numerous sites and times where prevalence was low, but density was high (Figure 2). Overall 31% of oyster samples were positive for *T. gondii*.

### 2.2. Cryptosporidium parvum Prevalence and Densities

**Figure 3:**
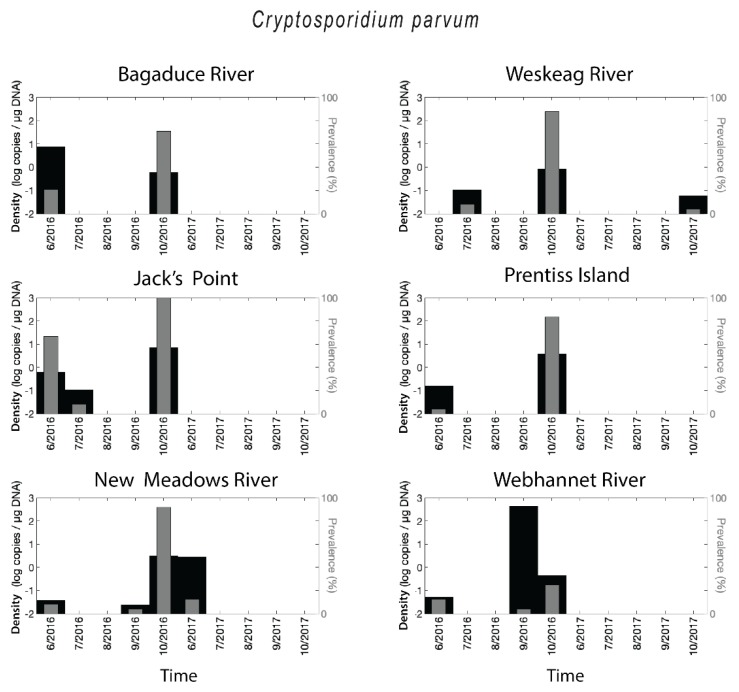
Spatial-temporal patterns for *Cryptosporidium parvum*. Retention intensity (as log of copies per µg DNA, shown as black bars) and parasite prevalence (as a percentage of oysters infected, shown as grey bars) for each sampling site, for each sampling period.

*Cryptosporidium parvum* DNA was detected at each of the six sites (Figure 3). The highest prevalence for *C. parvum* corresponded to October 2016 for five of the six sites, with values reaching 100% (Jack’s Point, DRE). This finding appears to have been a distinct, synchronous event across the Midcoast region of Maine, with the only excluded site being the southern Webhannet River site. Other than this event, the prevalence was generally low to zero, with only one other site and date where prevalence surpassed 50% (Jack’s Point, June 2016). In 2017 *C. parvum* was almost absent. When the parasite was present, *C. parvum* densities were in the range of less than 0.1 up to 423 copies/µg DNA for *C. parvum*. High levels of retention generally corresponded with the October 2016 event, but in a few instances (e.g., Bagaduce River, June 2016; New Meadows River, June 2017; Webhannet River, November 2016), retention was high despite low prevalence (Figure 3). Overall, 10% of oyster samples were positive for *C. parvum*.

### 2.3. Statistical Analysis

**Figure 4:**
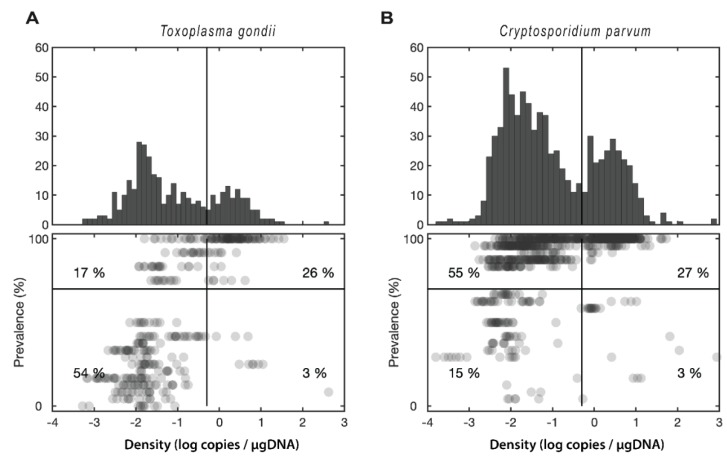
Distribution of prevalence versus intensity for the sampled sites. (**A**) *Toxoplasma gondii*. (**B**) *Cryptosporidium parvum*. Upper panel shows a histogram of parasite densities (as copies per µg DNA, log scale). The vertical line marks the split in the bimodal distribution. Lower panel shows a scatter plot of parasite densities versus prevalence (percentage of oysters infected at a given site/time). The numbers indicate the percentage of points that fall within each quadrant.

Prevalence and densities (log scale) were correlated with each other at a statistically significant level, though there was some nuance to these patterns. For *T. gondii*, the correlation was significant but fairly low (*r^2^* = 0.13, *p* < 0.001), reflecting the fact that the majority of samples (55%) fell in the low-intensity-high-prevalence quadrant (Figure 4a). For *C. parvum*, the correlation was higher (*r^2^* = 0.50, *p* < 0.001), primarily reflecting the strong bimodal distribution of retention, with nearly all samples (80%) having intensity and prevalence either both high or both low (Figure 4b).

**Figure 5:**
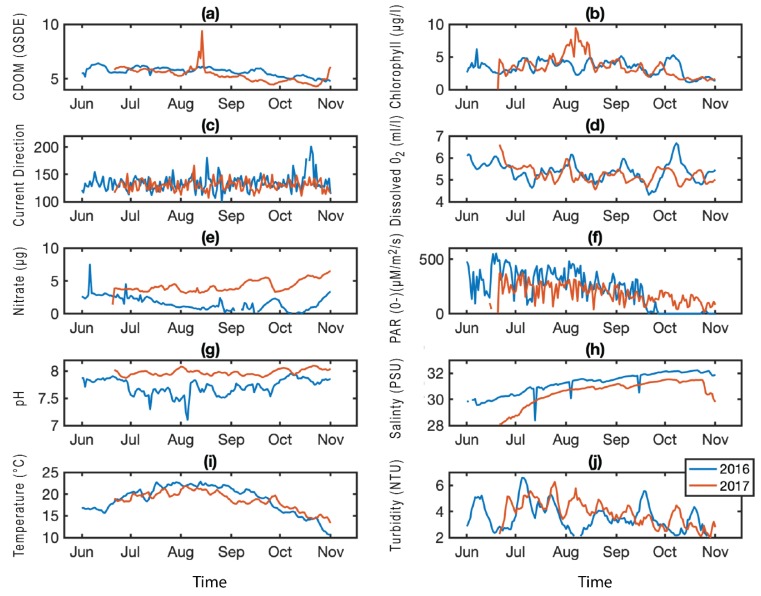
Prentiss Island environmental parameters. Averaged daily means for carbon dissolved organic matter (CDOM) (**a**), chlorophyll (**b**), current direction (**c**), dissolved oxygen (**d**), nitrate (**e**), photosynthetically active radiation (PAR) (**f**), pH (**g**), salinity (**h**), temperature (**i**), and turbidity (**j**) from a buoy located near the Prentiss Island (DRE) sampling site; data from 2016 (blue) and 2017 (red).

Statistical analysis of environmental data did not identify clear drivers of retention, but there were some notable significant patterns. The difference in *T. gondii* retention between 2016 and 2017 matched an environmental shift in the current direction, nitrate, and photosynthetically active radiation (PAR), all of which had significant differences (*p* < 0.05, *t*-test) between 2016 and 2017 measurements (Figure 5). Current direction and nitrate, measured near the Prentiss Island site and averaged into monthly values, correlated with *T. gondii* prevalence across the ten measurement times at Prentiss Island (current direction, *r*^2^ = 0.36, *p* = 0.06; nitrate, *r*^2^ = 0.70, *p* = 0.003). The occurrence of *C. parvum* was infrequent enough at the Prentiss Island site that statistical analysis was not possible. The one major retention event—October 2016—corresponded with the month of highest dissolved oxygen measurements as well as a shift in the current direction, from southeastward to southward, not seen at any other time (Figure 5).

## 3. Discussion

As more countries conduct surveys for protozoan parasites in bivalves intended for human consumption, the concern about the identification of human pathogens is also increasing [1,14]. The presence of *C. parvum* in bivalves from the East Coast of the United States was previously reported in the eastern oyster from 11 sites in the Chesapeake Bay with more than 80% of samples positives [24]. In that previous study, the authors used a combination of immunofluorescent antibody and in situ hybridization to quantify *C. parvum* oocysts in *C. virginica*. Interestingly, several of the oysters had a significantly higher number of viable oocysts than the rest of the pool and oocysts were identified in both the hemolymph and gills [24]. *Mya arenaria*, *Geukensia demissa*, and *Mytilus edulis* from Orchard Beach in New York were also positive for *C. parvum* using a PCR-based assay (1% and 50% prevalence for the two positive sites), the DNA was identified mostly in the gills and the foot, and the prevalence of *C. parvum* assemblage varied in the examined species even when they were collected within a short distance from one another [25]. *C. parvum* has also been reported in *M. arenaria* from the St. Lawrence River Estuary in Québec (Canada); a pool including foot, muscle, gonad, mantle, and intervalvular water was used for purification of the oocyst in a sucrose gradient, and the diagnostic was performed by an indirect immunofluorescent assay [26]. Recently, *T. gondii* has been associated with stranded Beluga whales in the same Canadian Province with necropsy results indicating toxoplasmosis as cause of death for one individual [27]. Previous studies from our group in 2014 detected for the first time DNA from both protozoan parasites in oysters from Maine (three sites for *C. parvum* and two for *T. gondii*). Here, we conducted a qPCR-based assay survey over 2 years, with focused sampling during the warm months of the year (June through October) to establish the retention dynamic of these two protozoan parasites in oysters from Maine. DNA from both *T. gondii* and *C. parvum* was detected in oysters from all the sites at some point during the survey. Interestingly, the highest prevalence for *T. gondii* corresponded to Jack’s Point and Prentiss Island in the DRE, the estuary that is home to 85–90% of the Maine oyster production. In all sampled sites the prevalence for *T. gondii* was much lower in 2016 than in 2017. This shift between 2016 and 2017 was also apparent in the parasite density values, although there were numerous sites and times where prevalence was low, and density was high. Since the source of oyster seed remains constant through the growing seasons in the DRE, it appears that these differences in the prevalence would be related to the source of the pathogen rather than the condition of the oysters. Similarly, *C. parvum* DNA was identified in the six sites sampled, although, with the exception of October 2016 for five of the six sites, the prevalence was generally low to zero, with only one other site and date where prevalence surpassed 50% (Jack’s Point, June 2016); in 2017 *C. parvum* was almost completely absent. Similar to the case of *T. gondii*, rather than changes in the ability of oysters to retain *C. parvum* DNA, the results likely reflect a sudden discharge of the protozoan into the environment. Genotyping of the parasite combined with land-based studies would have helped to pinpoint the origin of the DNA; however, this was outside the scope of the present study.

The quality of bivalve mollusks is closely related to the sanitary conditions of surrounding waters where they are cultivated [28]. Rainfall, especially during stormy events, can result in the mobilization of fecal material from land fields and farms, with subsequent discharge into rivers, increasing the concentration of fecal pathogens that are concentrated by filter-feeders and later transmitted to humans and marine mammals through the food chain [29,30,31,32,33,34,35,36,37]. The ability of shellfish to bioaccumulate, retain, and depurate microbial enteropathogens appears to be species-dependent [35]. Interestingly, in a few sites (e.g., New Meadows River and Webhannet River, June 2016 for *T. gondii*; Webhannet River, September 2016 for *C. parvum*), densities were high in some samples despite the low prevalence, indicating that some individual oysters might be more prone to concentrating the protozoans.

Experiments conducted in aquaria indicate that *T. gondii* and *C. parvum* oocysts can be retained in the bivalve for long periods in the gastrointestinal tract, gills, hemolymph, and intervalvular liquid [38,39,40,41,42,43,44]. It has been reported that *C. parvum* recovered from environmental samples retain viability [45], and although salinity affects the viability of *C. parvum* oocyst, they remain active for up to four weeks in estuarine conditions [33]. An approximation of the parasite load of wild mussels collected in *C. parvum* positive sites indicated that each shellfish transports between 6 and 1000 oocysts [46,47]. Some studies indicate that the amount of viable *C. parvum* oocysts is not related to oyster size or the level of fecal coliforms at the sampling site and that, although oysters are frequently contaminated with oocysts, the levels of viable oocysts may be too low to cause infection in healthy individuals [24]. Here 55% of the *T. gondii* infected oysters fell in the low-intensity but high-prevalence category while for *C. parvum*, nearly 80% had intensity and prevalence that were either both high or both low. Interestingly, DNA metabarcoding of microbial eukaryotes in water samples collected from the same areas did not reveal the presence of either protozoan parasite outside of oyster hosts (N.D.M., N.R.R., P.D.C., J.A.F.R., unpublished data). The presence of sporulated *T. gondii* oocysts in commercial green-lipped mussels (*Perna canaliculus*) from New Zealand has been recently reported via Reverse Transcriptase-PCR (RT-PCR) [48]; but whether the oocysts of both *T. gondii* and *C. parvum* could have excysted remains an open question. We used species-specific qPCR assays to target the presence and abundance of protozoan DNA in oyster tissues. As only sporulated *T. gondii* and *C. parvum* oocysts can be infectious, an RT-PCR targeting a sporozoite-specific marker (e.g., SporoSAG for *T. gondii*) [48] from active cells would be required to assess the potential risk of infection for human consumption. In the case of *C. parvum*, even if the excystment happens, the complexity of this parasite [49] makes infection from oyster cells very unlikely. Indeed, the latest data available for Maine (2007) reports a total of 56 *C. parvum* cases in the state. This translates into a per capita rate that is greater than the US national per capita rate, but could be explained by the rural and farming characteristics of the state. The small outbreaks reported in July of 2007 involved transmissions that occurred in school and family settings (Maine Center for Disease Control & Prevention). We have not been able to uncover any statistics for toxoplasmosis, which is a Reportable Disease or Condition in the state of Maine. A future crucial step for identifying the infection sources and evaluating the public health potential of the parasites to both humans and animals would be genotyping both protozoans. Genotyping of *Cryptosporidium* spp. is performed by sequencing the 60 kDa glycoprotein gene (*gp60*) or sequencing the entire genome [50,51]. In the case of *T. gondii*, there are more than 50 genetic markers for genotyping [52,53]. Our survey relied on qPCR detection of DNA extracted from oyster tissues, and the results could well be an indication that *T. gondii* oocysts are also passing through the bivalve [54,55]. Establishing the risk of *Toxoplasma* infection from eating raw bivalves [48] would require the development and validation of methods for the detection and survival/infectivity assays to enable robust risk assessments and implementation of control measures [2].

The Gulf of Maine is experiencing accelerated rates of climate change relative to other locations [56,57,58]; it is quite likely that the climate-driven changes affect the precipitation regime and consequently, the intensity and frequency of runoff events. However, direct linkages between climate change and both the prevalence and spread of waterborne parasites remain unclear and will require significant research efforts to understand. The two parasites examined in this study appeared to respond differently to environmental conditions. Previous work has shown that an oyster infected with one parasite can be more susceptible to other infection [23]. In this study, there was no significant correlation of infection intensity between the two parasites (*r* = –0.12, *p* = 0.18). More notably, the proportion of oysters infected by both parasites (0.76%) was far lower than the expected value from chance alone (3.2%), if infection by each parasite were random independent events. This finding implies that the conditions conducive to the two infections were to some degree, non-overlapping within this two-year survey. 

## 4. Materials and Methods 

### 4.1. Collection of Tissue Specimens and DNA Extraction 

*Crassostrea virginica* specimens were collected monthly between June and October of 2016 and 2017, were obtained from shellfish farmers located at six sites along the Maine coast: Bagaduce River (Brooksville), Weskeag River (Thomaston), Jack’s Point and Prentiss Island in the DRE (Lincoln), New Meadows River (Bath/New Meadows), and Webhannet River (Wells) (Figure 1). Upon arrival at the laboratory, the oysters were measured and weighed, notched for hemolymph extraction and immediately processed or stored at 4 °C for next day processing. Hemolymph was withdrawn (100–300 µL) from the anterior adductor muscle using an 18 gauge needle, fixed with Glutaraldehyde (0.2–1.5%) on ice, and stored at −80 °C for further analysis (data not shown).

Twenty-four oysters from each sampling site and collection date were individually dissected (n = 1440). For each oyster, rectum, gill, and mantle subsamples were collected and pooled (50–100 mg wet weight of total tissue/pool), and DNA was extracted using a commercial kit (Omega Biotek E.Z.N.A. Tissue Kit, Norcross, GA, USA). DNA concentration and purity were estimated with a Nanodrop^TM^ 2000 spectrophotometer and a Qubit 2.0 fluorometer (ThermoFisher Scientific, Waltham, MA, USA). The DNA samples, to facilitate the manual high throughput diagnostic, were diluted and aliquoted to contain 200–600 ng of DNA in 3 µL and stored at −20 °C until testing by qPCR.

### 4.2. qPCR Assays

For each sample, we ran qPCR-based assays specific for *T. gondii* based on the Internal Transcribed Spacer 1 (ITS1) region of the RNA gene cluster (forward primer 5′-GAT TTG CAT TCA AGA AGC GTG ATA GTA-3′; reverse primer 5′-AGT TTA GGA AGC AAT CTG AAA GCA CAT C-3′) [59] and *C. parvum* based on DnaJ-like protein (forward primer 5′-ACC TCA GAA GAA GAA ATC CTA-3′; reverse primer 5′-GCT CCT CAT ATG CCT TAT TG-3′, newly designed for this study). Primers and probes were evaluated using the IDT Primer Quest tool and AlleleID^®^ v.7 (PREMIER Biosoft), and purchased from Integrated DNA Technologies (IDT Inc. Coralville, IA, USA). The labeled probes were 5′-/TET/-CTG CGC TGC /ZEN/TTC CAA TAT TGG-/IABkFQ/-3′ and 5′-/6-FAM/-CCA ATC ACA/ZEN/GAA TCA TCA GAA TCG ACT GGT ATC-/IABkFQ/-3′ for *T. gondii* [59] and *C. parvum* [60], respectively. Both probes utilized dual-quenching with ZEN™ and Iowa Black^®^ (IDT Inc. Coralville, IA, USA) to decrease background fluorescence. All qPCR assays were performed using a Bio-Rad CFX96 optical unit with a C1000 thermal cycler (Bio-Rad, Hercules, CA, USA). Each assay was prepared using Bio-Rad skirted and semi-skirted 96 well plates and sealed with Microseal ‘B’ optical film (Bio-Rad). The total reaction volume was 25 µL per individual well consisting of 12.5 µL of PrimeTime^®^ Gene Expression Master Mix (IDT), 0.5 µL each of 10 µM forward and reverse primers, and 0.5 µL of 10 µM probe. PCR grade water was added at a volume of 8 µL, and 3 µL of DNA brought the total to 25 µL. DNA standards for qPCR were prepared from PCR-positive samples that were cloned, plasmid purified, linearized with NotI and diluted over five orders of magnitude (ranging from 10^6^ to 10^1^ copies per reaction). Exact copy numbers per microliter of the undiluted DNA standards were determined from the DNA concentration and the molecular weight of each cloned PCR product, and knowledge of the exact nucleotide composition of cloning vector plus PCR insert. Exact copy numbers for a particular dilution series varied slightly depending on the batch of a particular standard over the 2 year project. All samples and standards were run in triplicate to assess technical replication.

### 4.3. Environmental Data

An array of LOBO (Land/Ocean Biogeochemical Observatory) buoys is deployed along the coast of Maine as part of the University of Maine’s SEANET (Sustainable Ecological Aquaculture Network) project. One of these buoys was located near the Prentiss Island site from 2015–2018, collecting hourly data of multiple environmental parameters (carbon dissolved organic matter, CDOM (QSDE), chlorophyll (µg/L), current direction (degrees), dissolved oxygen (mL/L), nitrate (µM), photosynthetically active radiation, PAR (µM/m^2^/s), pH, salinity (PSU), temperature (°C), turbidity (NTU)). We used these data to examine possible environmental controls on the prevalence and intensity of parasite infections. For comparison with parasite data, environmental data were averaged to monthly means corresponding to oyster sample months. Basic exploratory statistical analyses were performed, including Pearson linear correlation coefficients and two-sample *t*-tests.

## 5. Conclusions

Our study represents a broad snapshot for these protozoan parasites in oysters from Maine; both origin (fecal contamination from wildlife or runoff from farmed land) and abilities of the oocysts to sporulate in oyster tissues were outside the scope of this study, and they remain unknown. Because of the sporadic detection, we were not able to assign a cause for the presence, and the spike observed. Consequently, it remains challenging to define high versus low-risk areas that would contribute to the development of sustainable and safe aquaculture. The qPCR assays used was useful to report the presence and abundance of protozoan DNA in oyster tissues. However, only, specific sporozoite-specific markers from active cells or physical isolation of oocysts and infectivity assays would provide a robust assessment of the potential risk of infection for human consumption as the DNA detected could represent oocysts passing through the bivalve. Method standardization for parasite detection should lead to better risk assessment of mollusks as a source of these waterborne pathogens and the development of safety regulations, similar to those existing for bacterial and viral pathogens encountered in the same mollusk species [61,62]. The intensity of infection varied from less than 0.001 up to 219 copies/µg DNA for *T. gondii* and from less than 0.1 up to 423 copies/µg DNA for *C. parvum* and it highlights the possible presence of considerable amounts of protozoans in individual oysters. We have established the baseline and dynamics of these two protozoan parasites in the oysters grown in Maine and propose that similar approaches be adopted for routine monitoring of these potential human health threats.
